# Ankle Dorsiflexor Function after Gastrocsoleus Lengthening in Children with Cerebral Palsy: A Literature Review

**DOI:** 10.3390/medicina58030375

**Published:** 2022-03-02

**Authors:** Nicholas Sclavos, Norine Ma, Elyse Passmore, Pam Thomason, H. Kerr Graham, Erich Rutz

**Affiliations:** 1 Department of Paediatrics, The University of Melbourne, Melbourne, VIC 3052, Australia; norinemaau@gmail.com (N.M.); kerr.graham@rch.org.au (H.K.G.); erich_rutz@hotmail.com (E.R.); 2 Hugh Williamson Gait Analysis Laboratory, The Royal Children’s Hospital, Melbourne, VIC 3052, Australia; elyse.passmore@rch.org.au (E.P.); pam.thomason@rch.org.au (P.T.); 3 Gait Lab & Orthopaedics, Murdoch Children’s Research Institute, Melbourne, VIC 3052, Australia; 4 Faculty of Engineering and Information Technology, The University of Melbourne, Melbourne, VIC 3052, Australia; 5 Medical Faculty, University of Basel, 4001 Basel, Switzerland

**Keywords:** cerebral palsy, equinus, gastrocsoleus lengthening, foot drop, gait analysis

## Abstract

*Background and Objectives*: Ambulant children with cerebral palsy can demonstrate persistent “foot drop” after successful gastrocsoleus lengthening (GSL) surgery for equinus deformity. This may be due to inadequate strength and/or selective motor control of the ankle dorsiflexor muscles. A procedure has been developed to reduce foot drop—Tibialis Anterior Tendon Shortening (TATS), to be performed in conjunction with GSL. However, it is currently unclear how ankle dorsiflexor function changes after surgery and which children could benefit from TATS. This review summarises changes in ankle dorsiflexor function after GSL for equinus, as reported in the literature. *Methods*: A search was performed of the Medline, Embase and PubMed databases from 1980 to 5 March 2021. Keywords included “cerebral palsy”, “equinus deformity”, “orthopedic procedures” and “gait analysis”. The search identified 1974 studies. Thirty-three cohort studies met the inclusion criteria for this review. *Results*: Twenty-two studies reported improvement in swing phase ankle dorsiflexion kinematics, after GSL. There was also evidence that clinical measures of ankle dorsiflexor strength improved after surgery. Four studies reported changes in selective motor control, with mixed results across the studies. *Conclusions*: There is good evidence that swing phase ankle dorsiflexion improves after GSL surgery. Although, there is limited evidence that this correlates with reduced foot drop or diminished need for an ankle-foot orthosis. Future research should be prospective, randomised, include a large sample size, and should focus on identifying the optimal candidates for TATS.

## 1. Introduction

Children with cerebral palsy (CP) develop musculoskeletal deformities secondary to a non-progressive upper motor neuron lesion during early development [1,2]. The most common of these deformities in ambulant children with CP is equinus [3,4]. This is a fixed contracture of the calf muscles (gastrocnemius or gastrocnemius and soleus), causing increased ankle plantarflexion throughout the entire gait cycle [5]. Equinus may be unilateral or bilateral, dependent on whether a child has unilateral or bilateral spastic cerebral palsy (USCP or BSCP) [6]. Management of fixed equinus is by surgical lengthening of the gastrocsoleus muscle-tendon unit (MTU), using a variety of techniques [7,8,9]. This may occur as a single-level surgery, or as part of multi-level surgery (MLS), targeting other areas of musculoskeletal deformity [10,11,12,13].

The various gastrocsoleus lengthening (GSL) techniques are classified into three groups, based on the anatomy of the gastrocsoleus MTU [8,9]. Zone 1 is from the gastrocnemius muscle origin, at the femoral condyles, to the end of the medial gastrocnemius muscle belly. Zone 2 is from the termination of the medial gastrocnemius muscle belly to the end of the soleus muscle fibres. Zone 3 is the Achilles’ tendon [8,9]. Zone 1 surgery is the most conservative and in general should be utilised in the management of equinus in children with BSCP [7]. Zone 3 surgery is useful for more severe equinus, often seen in children with USCP, and should almost never be used in children with BSCP [7]. This difference in management highlights a possible distinction in the underlying neuromuscular pathology amongst the two topographic subgroups of children with CP. This is a recognized area for future research [14].

In GSL surgery there is a risk of both ‘over-correction’ and ‘under-correction’ [7,15,16]. Three-dimensional gait analysis (3DGA) is the gold standard for planning surgery in ambulant children with CP and for assessing outcomes [14,17,18]. Kinematic improvements have been reported during the stance phase of gait in short- and long-term studies, in children with USCP and BSCP [13,19,20,21]. In contrast, there is less understanding of the effects of GSL on the swing phase of gait [22,23]. During the normal swing phase, the ankle dorsiflexor muscles, primarily tibialis anterior, contract to lift the foot, for the forefoot and toes to clear the ground [24]. This requires both adequate strength and selective motor control (SMC) [22,25]. Whilst GSL corrects the fixed contracture of the plantar flexors of the foot and ankle, it does not necessarily improve the function of the dorsiflexors [22,23]. This can cause foot drop, where the ankle remains in equinus during the swing phase. This can cause clinical symptoms such as catching of the toes, tripping and falling [22,23,26]. Many children with persistent foot drop after GSL are reliant on an ankle–foot orthosis (AFO) to optimize gait function [22,23,27].

A surgical procedure, known as Tibialis Anterior Tendon Shortening (TATS), has been described by Rutz et al. in 2011 [28]. It was theorised that the tibialis anterior muscle becomes elongated and weakened with chronic equinus deformity [28]. This procedure, performed in combination with GSL, may improve the function of the tibialis anterior muscle and hence improve ankle dorsiflexion during the swing phase of gait [28]. This has the potential to reduce the frequency of foot drop and obviate the need for long-term AFO use [29,30]. Retrospective cohort studies of the effectiveness of TATS have been published [28,29,31]. However, there is currently no clear understanding of which children will experience post-operative foot drop and therefore who may benefit from TATS [28,31]. It is by understanding how the function of ankle dorsiflexor muscles change, after GSL for equinus, that clinicians may be able to appropriately identify candidates for TATS. The aim of this review is to summarize the literature on the function of the ankle dorsiflexor muscles after GSL, in ambulant children with CP.

## 2. Materials and Methods

A search was performed via the Medline (Ovid), Embase (Ovid) and PubMed databases on 5 March 2021, dating back to 1980. The search strategy was developed by the authors, in conjunction with a medical librarian at The Royal Children’s Hospital, Melbourne. The following MeSH terms were used in various combinations for the Medline search: ‘cerebral palsy’, ‘equinus deformity’, ‘orthopedic procedures’, ‘tenotomy’, ‘neurological gait disorders’, ‘gait analysis’, and ‘treatment outcome’. The Medline search strategy was adapted for use in the other databases. The full search strategy, including key terms and Boolean operators, for all three databases, is provided in the supplementary material. All results were limited to English and those involving the paediatric or adolescent population.

The inclusion criteria for this review were cohort studies reporting original data on swing phase outcome measures after GSL, in children with CP. Children were defined as those aged 18 years or younger. References were excluded if they also included diagnoses other than CP or only included an adult population. Conference abstracts, case reports and scoping reviews were also excluded. Three additional studies were identified manually via the reference lists of the extracted articles from the database search. Data, including demographics, topographical classification and relevant post-operative swing phase outcomes, were extracted from each of the included references.

## 3. Results

**Figure 1:**
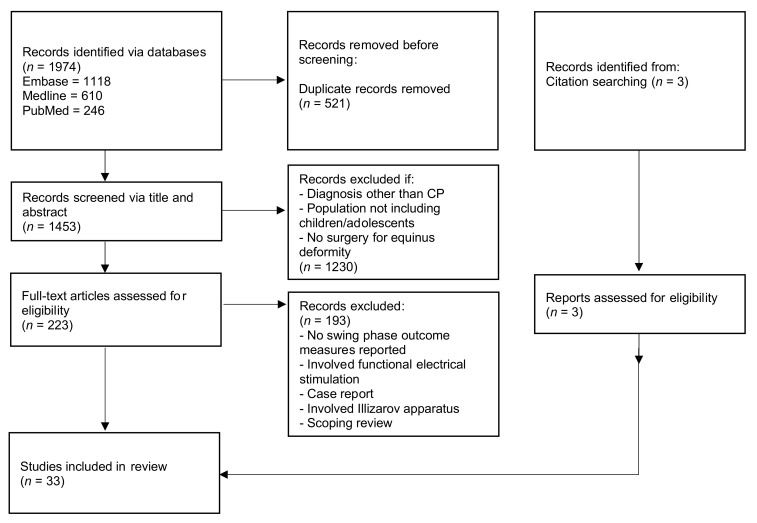
PRISMA diagram.

The search strategy identified a total of 1974 articles (610 via Medline, 1118 via Embase and 246 via PubMed). There were initially 521 duplicates removed. One author (N.S.) screened each title and abstract, and a further 1230 articles were removed as they were not relevant to the topic. The remaining 223 articles were screened via full text independently by the authors (N.S., E.R.). Thirty-three articles appropriately met the inclusion criteria and were included in this review [11,12,19,20,21,22,23,25,28,29,30,31,32,33,34,35,36,37,38,39,40,41,42,43,44,45,46,47,48,49,50,51,52]. Further details regarding conditions for exclusion and the final number of included studies are represented by the PRISMA diagram in Figure 1.

All 33 studies involved GSL for the management of equinus deformity in children or adolescents with CP [11,12,19,20,21,22,23,25,28,29,30,31,32,33,34,35,36,37,38,39,40,41,42,43,44,45,46,47,48,49,50,51,52]. Four studies also included data from adults [28,29,30,43]. These were deemed appropriate for this review as a majority of the cohorts included participants under 18 years of age. Twenty-nine studies [11,12,19,20,21,22,23,25,32,33,34,35,36,37,38,39,40,41,42,43,44,45,46,47,48,49,50,51,52] focused on GSL, either used as an isolated procedure or as part of MLS (Table S1, Supplementary Materials). Four studies [28,29,30,31] investigated outcomes of TATS, used in combination with GSL (Table S2, Supplementary Materials). All were cohort studies, reporting original data [11,12,19,20,21,22,23,25,28,29,30,31,32,33,34,35,36,37,38,39,40,41,42,43,44,45,46,47,48,49,50,51,52]. Seven were prospective studies [12,25,30,37,48,49,50]. Nineteen were retrospective studies [19,21,22,23,28,29,31,32,33,34,35,38,39,40,43,44,47,51,52]. Within the remaining seven studies it is unclear whether they were prospective or retrospective [11,20,36,41,42,45,46].

### 3.1. Swing Phase Ankle Kinematics

All of the GSL studies, except that by Reimers [25], included gait kinematics [11,12,19,20,21,22,23,25,32,33,34,35,36,37,38,39,40,41,42,43,44,45,46,47,48,49,50,51,52]. Twenty-three studies reported swing phase kinematic data: maximum and/or mean ankle dorsiflexion range of motion (ROM) [11,12,19,20,22,23,32,33,35,38,39,40,41,43,44,45,46,47,48,49,50,51,52]. Twenty-two studies found statistically significant improvement in ankle dorsiflexion ROM during swing phase, after GSL (*p* < 0.05) [11,12,19,20,22,23,32,33,35,38,39,40,41,43,44,45,46,48,49,50,51,52]. Sung et al. [47] reported on 29 children with BSCP, in whom maximum dorsiflexion increased from 9.8° pre-operatively, to 14.5° at one-year follow-up (*p* = 0.47). Children in this cohort received either Zone 1 or Zone 3 surgery and surgery type was not included as a variable for statistical analysis. At the ten-year follow-up, maximum dorsiflexion had decreased to a mean of 7.1° [47]. Yngve and Chambers [52] found no significant improvement in maximum dorsiflexion in a subgroup of children in their cohort that received Zone 2 surgery (three USCP, 19 BSCP). In this group, maximum dorsiflexion increased from −3.0° to 2.0° (*p* = 0.4). There was a significant improvement found in those children who received Zone 1 surgery (11 USCP, 16 BSCP, *p* < 0.001) [52].

Two studies reported stratified outcomes for children with USCP and BSCP [39,51]. Lofterød and Terjesen [39] reported a greater improvement in maximum ankle dorsiflexion in children with BSCP than those with USCP. There was a mean improvement of 20.6° in children with BSCP (*p* < 0.001) and 16.3° in children with USCP (*p* = 0.013) [39]. This study included a small cohort (six USCP, nine BSCP). Tylkowski et al. [51] likewise reported greater improvement in maximum dorsiflexion in children with BSCP. There was a mean improvement of 13.4° in children with BSCP (*p* < 0.001) and 12.0° in children with USCP (*p* = 0.001) [51]. This study also included a small cohort (13 USCP, 14 BSCP). Neither study included between-group statistical analysis. The results of these two studies may suggest that children with BSCP have greater improvement in maximum dorsiflexion ROM during the swing phase, than those children with USCP.

Lofterød et al. [22] reported the frequency of persistent foot drop after GSL. In their study, foot drop was defined as maximum ankle dorsiflexion during the mid-swing phase below two standard deviations of the mean value, from a typically developing population. Lofterød et al. reported 19 of 40 operated limbs (47.5%) demonstrated foot drop after GSL, despite satisfactory correction during the stance phase. There was no significant association between post-operative foot drop and pre-operative physical examination measures, type of gait pattern, type of cerebral palsy, and type of GSL surgery [22].

### 3.2. Electromyography

Four studies included dynamic electromyography measures relevant to the swing phase [23,33,35,37]. None of the studies found any significant change in gastrocnemius, soleus or tibialis anterior muscle activation during the swing phase, after GSL. Davids et al. [23] were able to identify some degree of ankle dorsiflexor activation prior to surgery, which was maintained, but not improved, after surgery. Davids et al. [23] and Granata et al. [37] both found abnormal co-activation of the gastrocnemius and tibialis anterior muscles during the first half of the swing phase, both before and after GSL. Baddar et al. [33] reported less activity of the tibialis anterior muscle during the swing phase after GSL surgery. Although, this was not deemed significant due to high variability within the data [33]. The results of these four studies provide evidence that swing phase tibialis anterior muscle activation does not change after GSL [23,33,35,37].

### 3.3. Physical Examination Measures

The physical examination measures relevant to swing phase ankle dorsiflexion function are active ankle dorsiflexion ROM (knee flexed and extended), ankle dorsiflexion manual muscle strength and SMC. Thirteen of the GSL studies reported these measures [11,12,19,20,22,23,25,32,36,38,43,45,46]. Seven studies assessed active dorsiflexion ROM, all finding significant improvement after GSL [11,12,20,22,32,45,46]. Eight studies reported ankle dorsiflexion strength [11,12,19,20,23,25,36,46]. Three studies included children who had typical ankle dorsiflexion strength before GSL, which was maintained after surgery [11,12,20]. Four studies reported significant improvement in ankle dorsiflexion strength after GSL [19,23,25,46]. Galli et al. [36] found no statistically significant change in dorsiflexion strength. This was in a relatively small cohort of 20 children with CP (eight USCP, 12 BSCP).

Four studies included measures of SMC, with mixed results [22,23,36,38]. Lofterød et al. [22] and Davids et al. [53] reported significant improvement in SMC in their cohorts. Lofterød et al. [22] assessed SMC via the Boyd and Graham SMC Scale [54]. Davids et al. [23] used a four-point scale from the SMALnet protocol [53]. Galli et al. [36] and Kay et al. [38] both found no significant post-operative improvement in SMC. Both studies assessed SMC via a three-point scale and it is unclear whether this is sensitive enough to appropriately identify significant changes in SMC after surgery [36,38]. There was no between-group comparison of SMC in children with USCP and BSCP, in these studies [22,23,36,38]. Currently, there are conflicting results in the literature regarding SMC improvements after GSL for equinus.

### 3.4. Ankle–Foot Orthosis Use

Dreher et al. [19] reported reduced long-term rates of AFO-use after GSL, in their cohort of 44 children with BSCP. Pre-operatively, 59% (26/44) of children required AFO-use for mobilising [19]. All children required AFO-use at the one-year follow-up [19]. This reduced to 32% (14/44) at the three-year and 25% (11/44) at the nine-year follow-up periods [19]. Precise criteria for AFO-use/no AFO-use were not specified.

### 3.5. Tibialis Anterior Tendon Shortening

Four studies investigated the outcome of GSL, used in combination with TATS [28,29,30,31]. Three were retrospective [28,29,31] and one prospective [30]. Two studies reported maximum ankle dorsiflexion swing phase kinematics [29,30]. Tsang et al. [30] demonstrated an improvement in children with both USCP and BSCP, as reported via the Movement Analysis Profile [55], with no between-group mean difference (10.67° vs. 12.62°, *p* = 0.709). This cohort included 13 children with USCP and 13 with BSCP. At follow-up (mean 17.1 months), 50% of limbs (14/28) had “improved ease of AFO fitting” and 18% (5/28) no longer required an AFO [30]. The children in this study who did not have improved ankle dorsiflexion in the swing phase also had poor pre-operative SMC of tibialis anterior. Tsang et al. concluded that children who do not have selective activation of tibialis anterior pre-operatively should not undergo the TATS procedure.

Dussa et al. [29] compared maximum ankle dorsiflexion between children who received GSL and those that received GSL in combination with TATS (GSL/TATS). Amongst the children with USCP, 12 received GSL and 12 received GSL/TATS [29]. The maximum dorsiflexion in the swing phase improved by 13.7° in the GSL group and 11.7° in the GSL/TATS group [29]. Both improvements were significant and there was no between-group difference. In the group with BSCP, 11 received GSL and 9 received GSL/TATS. Improvements were 6.0° in the GSL group and 13.0° in the GSL/TATS group, although both improvements were not significant [29].

Dussa et al. [29] also reported no significant improvement in ankle dorsiflexor strength in either the GSL or the GSL/TATS groups, in both children with USCP and BSCP. Similar findings were also reported by Rutz et al. [28] and Klausler et al. [31]. This contradicts the original rationale for TATS [28] and highlights conflicting opinions in the literature. Dussa et al. concluded that there were no significant differences in clinical or kinematic outcome measures between those children that received TATS and those that did not. Furthermore, they believed children with good pre-operative ankle dorsiflexor function would not benefit from TATS [29]. It must be noted that this study also included some adults with CP (mean age 16.8 years, range 5–29 years).

## 4. Discussion

There is currently limited understanding of how the function of the ankle dorsiflexor muscles change after GSL surgery for equinus [22,23]. Understanding these changes is important to identify which children with CP might benefit from TATS. In 1990, Reimers was the first to report objective evidence for improvement in antagonist function after surgical lengthening of the contracted agonist, in children with CP. [25,56]. Since that time, there have been conflicting opinions in the literature [22,23,35]. Whilst many studies have focused on changes during the stance phase of gait, there is published evidence that GSL improves ankle dorsiflexion during the swing phase of gait, based on kinematic data [11,12,19,20,22,23,32,33,35,38,39,40,41,43,44,45,46,48,49,50,51,52]. There is also evidence that ankle dorsiflexion strength [19,23,25,46] and SMC [22,23] may improve. However, it is unclear whether these improvements translate to fewer children with persistent foot drop, better function, or reduced long-term AFO-use after surgery.

Lofterød et al. [22] investigated pre-operative predictive factors for foot drop after GSL. In their study, no significant correlation was identified between pre-operative variables (including passive ankle ROM, dynamic muscle length, SMC, functional classification of CP, type of gait pattern, type of CP and type of surgery) and persistent foot drop. The kinematic results indicated that a pre-operative maximum ankle dorsiflexion in the initial swing phase of less than −42° was associated with persistent foot drop after GSL [22]. Selective motor control was measured in 19 of the 40 operated limbs and good pre-operative SMC was found to predict normal post-operative swing phase function [22]. This suggested that GSL may unmask adequate ankle dorsiflexor function, if it is present pre-operatively. Although, the challenge in identifying adequate pre-operative SMC is that many children with CP, particularly those with USCP, have severe equinus and spasticity precluding accurate assessment.

From the four studies that included EMG data, there was no evidence that the activation of tibialis anterior improved after GSL [23,33,35,37]. Dreher et al. [35] concluded that despite the effects of GSL to reduce muscle tone, improve joint mobility and overall gait pattern, there was no change to the patterns of muscle activation observed in children with CP. The conclusion from previous studies was that whilst surgery was effective in lengthening the gastrocsoleus MTU, the abnormal muscle activation during gait, may not change [23,33,35,37]. This supports the conclusions of Lofterød et al. [22] that GSL may unmask adequate ankle dorsiflexor function, only if it is present pre-operatively.

Adequate improvement in swing phase ankle dorsiflexor function may be reflected by reduced rates of AFO-use, after GSL. Dreher et al. [19] demonstrated reduced long-term rates of AFO-use in their cohort of 44 children with BSCP. They reported 59% of children required AFOs pre-operatively and 25% at the nine-year post-operative follow-up. However, the authors of this study did not define their criteria for ‘AFO-use’ [19]. Some children with CP require AFOs for all mobility and others only for walking long distances. Rates of AFO-use alone may not be an accurate reflection of swing phase ankle dorsiflexor function, after GSL.

Two previous studies have compared post-operative kinematic outcomes between children with USCP and BSCP [39,51]. Both Lofterød and Terjesen [39] and Tylkowski et al. [51] reported a greater improvement in swing phase maximum ankle dorsiflexion in children with BSCP, than those with USCP. However, both studies demonstrated significant improvement in both topographical subgroups of children [39,51]. Both studies had small cohort sizes and neither included statistical analysis of between-group differences. Neither study included electromyography, physical examination outcome measures or information regarding post-operative AFO-use [39,51].

A major limitation of previous gait studies is small cohort size, with many studies including cohorts of fewer than 20 participants [12,20,21,31,33,36,39,40,41,43,48,50]. Due to this, there are often not enough participants for statistical comparison between the two principal topographical subgroups of children with CP, and analysis is undertaken on a mixed cohort of children with USCP and BSCP [22,23,25,28,29,30,31,32,34,36,38,39,42,43,48,51,52]. However, it is understood that these subgroups may respond differently to GSL, as children with USCP often demonstrate more severe equinus and require more extensive surgery [7,16]. It is important that future investigation stratifies these subgroups to identify between-group differences. There is evidence that children with USCP have less improvement in swing phase ankle dorsiflexion after GSL and these children may be appropriate candidates for TATS. This is an area for future research.

Previous studies support the effectiveness of TATS [28,29,30,31]. However, there are mixed opinions regarding the mechanism by which it may increase ankle dorsiflexion in the swing phase. There is evidence that ankle dorsiflexion strength may remain unchanged after TATS [28,29,31]. This contradicts the original rationale described by Rutz et al. [28]. Whilst improved ankle dorsiflexor strength and SMC would be the ideal outcome, improvements in swing phase function may originate from other mechanisms, such as a tenodesis effect [57]. Shortening the tibialis anterior tendon may hold the ankle in greater dorsiflexion at rest and increase ankle dorsiflexion during the swing phase of gait, without improving strength or SMC. This could explain the kinematic improvements in ankle dorsiflexion during the swing phase, reported in previous studies [29,30]. Dussa et al. [29] provide evidence that TATS does not reduce the risk of foot drop after GSL and may only have benefits in children with poor pre-operative ankle dorsiflexor function. However, prospective investigation of larger paediatric-only cohorts is required to further develop accurate methods of identifying appropriate candidates for this procedure.

A limitation of this review’s methodology is that whilst two reviewers were involved in the inclusion of studies, a single reviewer performed the quantitative synthesis of data. Another limitation is the quality of the literature itself. This includes a majority of small sample sizes, retrospective study designs and heterogeneous cohorts of children with CP. Whilst we have endeavoured to accurately compare the outcome measures of the 33 studies, it must be acknowledged that each cohort of children with CP vary significantly and comparison is not entirely reliable.

Future studies investigating ankle dorsiflexion function after GSL must include larger sample sizes, allowing for independent analysis of outcomes in children with USCP and BSCP. Due to the varying severity of musculoskeletal deformities observed in children with CP, it is important that these studies include more specific details on the severity of equinus deformity, gait pattern and motor function classification. For more robust evidence, prospective studies and randomised surgical trials involving TATS will be necessary.

## 5. Conclusions

Amongst those children who receive GSL for equinus, there is a need to further identify candidates for TATS, to minimise the burden of persistent foot drop, improve function and reduce long-term AFO-use after surgery. This review identified evidence that isolated GSL improves ankle dorsiflexion during the swing phase of gait. However, there is limited evidence that this improvement correlates with reduced rates of post-operative foot drop or reduced long-term need for an AFO. There is some indication that the function of the ankle dorsiflexor muscles improves after GSL, based on post-operative improvements in active ankle dorsiflexion ROM and manual muscle strength. Although, there is not enough reliable data that the SMC of the tibialis anterior improves. Future research should focus on identifying the optimal candidates for TATS. It should be prospective, randomised and involve a large sample size, including comparable groups of children with USCP and BSCP.

## Supplementary Materials

The following supporting information can be downloaded at: https://www.mdpi.com/article/10.3390/medicina58030375/s1, Table S1: Gastrocsoleus Lengthening Studies; Table S2: Tibialis Anterior Tendon Shortening Studies.
