# Supplementary material for: Ankle Dorsiflexor Function after Gastrocsoleus Lengthening in Children with Cerebral Palsy: A Literature Review
Source: Medicina (Kaunas). 2022 Mar 2;58(3):375. doi: 10.3390/medicina58030375 (PMC8955202; doi:10.3390/medicina58030375)
Supplement: Supplementary file 1 [file medicina-58-00375-s001.zip › medicina-1599221-supplementary.pdf]

Table S1. Gastrocsoleus Lengthening Studies.

Study	Aims	Retrospective/ Prospective	No. of Participants	Mean Age at Surgery in Years (Range)	Relevant Outcome Measures	Results
Adolfsen, S. E., et al. 2007 **[32]**	Outcomes of the medial hamstring lengthening, rectus femoris transfer, and GSL	Retrospective	10 USCP, 21 BSCP	8.5 (5-15)	Active ankle DF ROM (both knee flexed and extended), kinematics	- Significant improvement in active ankle DF ROM (knee flexed and extended) and in maximum ankle DF in swing kinematics
Baddar A et al. 2002 **[33]**	Length and excursion of gastrocsoleus tendon-unit in relation to knee and ankle kinematics	Retrospective	11 BSCP	6.1 (3.6-11.2)	EMG, kinematics	- Tibialis anterior had less relative activity in late stance and swing postoperatively, this was non-significant because of high variability<br>- Significant improvement in mean ankle DF swing kinematics
Chung, C. Y., et al. 2015 **[34]**	What is the recurrence rate of equinus after TAL and what are the risk factors?	Retrospective	72 USCP, 171 BSCP	7.8 (4.4 - 16.9)	Kinematics	- Analysis only based on recurrence of equinus, not total group
Davids, J. R., et al. 2011 [23]	Dorsiflexor function during swing phase (MLS and isolated surgery)	Retrospective	33 USCP, 20 BSCP	8.9 (range not provided)	SMC (Smalnet protocol), strength, kinematics	- Significant improvement in SMC, strength and in mean and maximum ankle DF in swing phase kinematics
Dreher, T., et al. 2013 **[35]**	Effects of calf muscle-tendon lengthening on muscle tone and activation pattern	Retrospective	42 BSCP	9.8 (6-16)	Kinematics, dynamic EMG	- Significant improvement in maximum ankle DF in swing kinematics<br>- No significant improvement in tibialis anterior activation in swing phase
Dreher, T., et al. 2012 [19]	Long term outcome of gastrocsoleus intramuscular aponeurotic recession (as part of MLS)	Retrospective	44 BSCP	9.8 (range not provided)	AFO-use, strength, kinematics	- AFO-use decreased from 59% pre-op to 25% at nine-year follow-up<br>- Significant improvement in strength and in mean and maximum ankle DF swing kinematics
Galli, M., et al. 2005 **[36]**	Objective evaluation of the effect of the modified Vulpius gastrocnemius lengthening technique on gait pattern	Unclear	8 USCP, 12 BSCP	8 (7-11)	Strength, SMC (0-2 scale), kinematics	- No significant improvement in strength<br>- No significant improvement in SMC<br>- Only kinematic graphs included, no data
Galli, M., et al. 2009 [20]	Long-term outcomes after modified Vulpius surgery (isolated surgery)	Unclear	12 BSCP	9.3 (5-14)	Active ankle DF ROM, strength, kinematics	- Significant improvement in active DF ROM, strength and in maximum ankle DF swing kinematics
Granata, K. P., et al. 2000 **[37]**	Effect of muscle-tendon lengthening on gait dynamics by analysing joint angular velocities and EMG data	Prospective	40 BSCP	8.3 (3.7 - 14.6)	Dynamic EMG, kinematics	- No change in tibialis anterior activation in swing phase<br>- Only kinematic graphs included, no data

Kay, RM et al. 2004 **[38]**	Outcomes of TAL and gastroc recession in ambulatory children with cerebral palsy	Retrospective	23 USCP, 32 BSCP	11.1 for Zone 1 group; 9.6 for Zone 3 group (no range provided)	SMC (0-2 scale), kinematics	- No significant change in SMC<br>- Significant improvement in mean ankle DF swing kinematics in total group
Lofterød, B. and T. Terjesen 2008 **[39]**	Local and distant effects of GSL; compare children with USCP and BSCP	Retrospective	6 USCP, 9 BSCP	8.8 (6-14)	Kinematics	- Significant improvement in maximum ankle DF swing kinematics in: (1) total group; (2) children with USCP; (3) children with BSCP
Lofterød, B., et al. 2009 **[22]**	Pre-op predictors for drop foot after MLS	Retrospective	16 USCP, 18 BSCP	9.3 (5-18)	Active ankle DF ROM (knee extended), SMC (Boyd and Graham SMC score), kinematics	- Significant improvement in active ankle DF ROM (knee extended), SMC and in maximum ankle DF in swing kinematics
Lyon, R., et al. 2005 **[40]**	Compare ankle, knee, hip kinematic data and temporal data, before and after TAL in children with BSCP	Retrospective	14 BSCP	9.1 (no range provided)	Kinematics	- Significant improvement in maximum ankle DF in swing kinematics
Park, C. I., et al. 2006 **[41]**	Local and distant effects of GSL surgery	Unclear	16 USCP	8.3 (3-16)	Kinematics	- Significant improvement in mean ankle DF swing kinematics
Patikas, D., et al. 2007 **[42]**	Changes in EMG patterns after MLS	Unclear	16 USCP, 18 BSCP	10.1 (6-16)	Kinematics, dynamic EMG (but no swing phase), kinetics	- Only kinematic graphs included, no data
Reimers, J. 1990 [25]	Antagonist muscle function after lengthening spastic calf muscles	Prospective	6 USCP, 46 BSCP	Median age 5 years (2.3-18)	Ankle DF Strength	- Significant improvement in strength
Rose, S. A., et al. 1993 **[43]**	Outcome of modified Baker technique (MLS)	Retrospective	5 USCP, 15 BSCP	6 (4-26)	Kinematics, EMG (not including tibialis anterior)	- Significant improvement in maximum ankle DF in swing kinematics
Saraph, V., et al. 2000 **[45]**	Outcomes of Baumann procedure (MLS)	Unclear	22 BSCP	12.6 (7.4-16.6)	Active ankle DF ROM (knee flexed and extended), kinematics	- Significant improvement in active DF ROM (knee flexed and extended) and maximum DF in swing phase kinematics
Saraph, V., et al. 2005 **[44]**	MLS outcome of children with BSCP after one-, two- and three-years	Retrospective	32 BSCP	11.1 (8.7 - 13.5)	Kinematics	- Significant improvement in maximum ankle DF in swing kinematics
Saraph, V., et al. 2002 [11]	Outcome of gait improvement surgery	Unclear	25 BSCP	13.6 (6 - 15.5)	Active ankle DF ROM, strength, kinematics	- Significant improvement in active ankle DF ROM and in maximum ankle DF swing kinematics<br>- Strength maintained (4/4 pre- and 4/4 post-op)
Steinwender, G., et al. 2001 **[46]**	Compare outcomes in children with BSCP, two groups: (1) dynamic equinus managed conservatively, (2) fixed equinus managed with GSL	Unclear	17 BSCP	14.4 (no range provided)	Active ankle DF ROM (knee flexed and extended), strength, kinematics	- Significant improvement in active ankle DF (knee and flexed and extended), strength and maximum ankle DF in swing kinematics

Sung, K. H., et al. 2013 **[47]**	Long- term outcome of MLS including Distal Hamstring Lengthening	Retrospective	29 BSCP	8.3 (5.4-16.3)	Kinematics	- No significant improvement in maximum ankle DF in swing kinematics and significantly worse at 10 year follow-up
Svehlik, M., et al. 2012 [21]	Long term outcomes Baumann procedure (MLS)	Retrospective	18 BSCP	11.5 (7-16.5)	Kinematics	- Only kinematic graphs included, no data
Svehlik, M., et al. 2008 **[48]**	Gait outcomes nine-months after soft tissue surgery	Prospective	2 USCP, 9 BSCP	9.1 (no range provided)	Kinematics	- Significant improvement in mean ankle DF in swing kinematics
Terjesen, T., et al. 2015 **[49]**	(1) Gait parameters and gait function one-year post MLS (2) Patient satisfaction (3) Additional orthopaedic surgery after MLS	Prospective	34 BSCP	5.6 (3-9)	Kinematics	- Significant improvement in maximum ankle DF in swing kinematics
Thompson, N., et al. 2010 **[50]**	Pilot study comparing outcomes of minimally invasive MLS and conventional MLS	Prospective study	10 BSCP	10.6 (7.11-13.9)	Kinematics	- Significant improvement in maximum ankle DF swing kinematics
Tylkowski, C. M., et al. 2009 **[51]**	Outcomes after isolated gastro-soleus lengthening, using instrumented gait analysis	Retrospective	13 USCP, 14 BSCP	11.4 (no range provided)	Kinematics	- Significant improvement in maximum ankle DF swing kinematics in both children with USCP and BSCP
Yngve, D. A. and C. Chambers 1996 **[52]**	Compare Vulpius (Zone 2) and Z-lengthening (Zone 3) procedures	Retrospective	Zone 2: 3 USCP, 19 BSCP; Zone 3: 11 USCP, 16 BSCP	No age provided	Kinematics	- Significant improvement in maximum ankle DF swing kinematics in Zone 3 group<br>- No significant improvement in maximum ankle DF swing kinematics in Zone 2 group
Zwick, E. B., et al. 2001 [12]	Outcome of gait improvement surgery using well defined criteria in children with BSCP	Prospective	17 BSCP	11.2 (5.7 - 16.4)	Active ankle DF ROM, strength, kinematics	- Significant improvement in active ankle DF ROM and maximum ankle DF swing kinematics<br>- Strength maintained (4/4 pre- and 4/4 post-op)

MLS = multilevel surgery, USCP = unilateral spastic cerebral palsy, BSCP = bilateral spastic cerebral palsy, SMC = selective motor control, DF = dorsiflexion, ROM = range of motion, CP = cerebral palsy, EMG = electromyography, TAL = tendino-achilles lengthening, GSL = gastrocsoleus lengthening

Table S2. Tibialis Anterior Tendon Shortening Studies.

Study	Research Question	Retrospective/ Prospective	No. of Participants	Mean Age at Surgery in Years (range)	Relavent Outcome Measures	Results
Dussa, C. U., et al. 2021 [29]	Compare GSL alone and GSL/TATS combined procedures	Retrospective	24 USCP, 20 BSCP	11.5 (6-29.0)	Strength, kinematics	- No significant improvement in strength following TATS<br>- Significant improvement in maximum ankle DF swing kinematics, no between group differences (GSL vs GSL+TATS)
Klausler, M., et al. 2017 **[31]**	Long-term outcome of combinated TATS and Zone 3 GSL (including single level and MLS)	Retrospective	12 USCP, 8 BSCP	14.9 (no range provided)	Strength, kinematics	- No significant improvement in strength in either children with USCP or BSCP<br>- No swing phase kinematic data reported
Rutz, E., et al. 2011 [28]	Short-term outcome of combinated TATS and Zone 3 GSL (including single level and MLS)	Retrospective	21 USCP, 8 BSCP	15.1 (no range provided)	Strength, kinematics	- No significant improvement in strength in either children with USCP or BSCP<br>- No swing phase kinematic data reported
Tsang, S. T. J., et al. 2016 [30]	Short term results of combined TATS and GSL procedures	Prospective	13 USCP, 13 BSCP	16.8 (10.3-34.5)	Kinematics	- Significant improvement in maximum DF swing kinematics<br>- No between groups differences in children with USCP and BSCP

TATS = tibialis anterior tendon surgery, GSL = gastrocsoleus lengthening, MLS = multilevel surgery, USCP = unilateral spastic cerebral palsy, BSCP = bilateral spastic cerebral palsy, DF = dorsiflexion, ROM = range of motion, TAL = tendo-achilles lengthening
